# Implementing Silent Disco Headphones in a Hospital Unit: A Qualitative Study of Feasibility, Acceptance, and Experience Among Patients and Staff

**DOI:** 10.1177/23779608211021372

**Published:** 2021-05-30

**Authors:** Lillian Hung, PhD, RN, Kevin Dahl, BMT, Gail Peake, Luka Poljak, Lily Wong, Jim Mann, LLD, Michael Wilkins-Ho, MD, Habib Chaudhury, PhD

**Affiliations:** 1 School of Nursing, University of British Columbia, Vancouver, British Columbia, Canada; 2 Willow 5, Older Adult Program, Vancouver General Hospital, Vancouver, British Columbia, Canada; 3 CEAN Community Engagement Advisory Network, Vancouver Coastal Health Authority, Vancouver, British Columbia, Canada; 4 Gerontology, Simon Fraser University, Burnaby, British Columbia, Canada

**Keywords:** dementia, chronic illnesses, music, mental health, technology

## Abstract

**Introduction:**

Music is so widely available and inexpensive in the modern world; it is a common option for stress reduction, comfort and enjoyment. Silent disco headphones are used among young people; however, no study has yet investigated whether it is feasible to use these headphones to support mental health and well-being among older people with dementia in hospital settings.

**Objective:**

The study’s main objective is to explore whether music delivered by silent disco headphones was feasible and acceptable to a sample of inpatients staying in an older adult mental health unit of a large urban hospital.

**Methods:**

We employed a video-ethnographic design in data collection, including conversational interviews and observations with video recording among ten patient participants in a hospital unit. A focus group was conducted with ten hospital staff on the unit.

**Results:**

Our analysis identified three themes that represented experiences of patients and staff: (1) perceived usefulness, (2) perceived ease of use, and (3) attitude. Patient participants reported the music delivered by the headphones brought positive benefits. Witnessing the positive effects on patients influenced the staff’s view of how music could be used in the clinical setting to support patients’ well-being.

**Conclusions:**

The music delivered by the silent disco headphones in an older adult mental health unit was found to be an acceptable and feasible intervention for patients. Leadership support is identified as an enabling factor in supporting technology adoption in the clinical setting. The findings can be used to inform practice development and future research.

Loneliness, depression, and anxiety are common symptoms for patients with dementia in care settings (Ørjasæter et al., 2018). In particular, loneliness has also been associated with hypertension, sleep problems, functional decline, and cognitive impairments (Musich et al., 2015). Unmet psychosocial needs are linked to behavioural symptoms of people with dementia (e.g., agitation, aggression and frustration) that may pose a risk to safety (Hung et al., 2017). Noise in the clinical environment may irritate and frustrate patients. Music and headphone technology could be useful tools to reduce patients’ behavioural symptoms and address unmet needs. The silent disco headphones can also provide a safe option of remote access to music therapy group in care settings as we are adapting to the new infection prevention procedures during and after the COVID-19 pandemic.

## Review of Literature

Research shows that restrictive clinical environments in hospitals and deprived meaningful engagement can aggravate patients’ vulnerability and behavioural symptoms (Cohen-Mansfield et al., 2012; Passmore et al., 2012; Sampson et al., 2014; Spencer et al., 2013). Noise can increase agitation and disturb sleep in patients with dementia (Kales et al., 2014). In a recent study, an older patient with dementia reported the need to get away from the stress of noise in the medical ward (Hung et al., 2017). The unfamiliar hospital environment can be anxiety provoking, with a large team of staff perform tasks against a backdrop of noises from alarms and machines, making the space disorientating (Featherstone et al., 2019; Hung et al., 2014). The particular needs of older patients with cognitive impairment are often poorly recognized by hospital staff. Collier et al. (2020) found much can be done in the hospital setting to meet patients’ fundamental needs if the team is supported to translate person-centred care from rhetoric to reality. When patient behaviors, such as agitation, are understood by staff as as communication of an unmet need, staff may be more likely to consider the underlying causes and take actions to meet the patient’s need (Handley et al., 2019).

Music has been researched for its potential to produce benefits for patients with dementia. A systematic review reported music therapy improves behavioral symptoms, anxiety and agitation (Gómez-Romero et al., 2017). Meta-analysis findings indicated that music therapy provides beneficial effects for people with depression (Aalbers et al., 2017). Tarr et al. (2016) described that music-based activities improve attention, allow patients to experience feelings of self-worth and achievement, and create a sense of togetherness. Other systematic reviews suggested that music reduces distress related to anxiety and improves everyday functioning (Garrido et al., 2017; Lee & Thyer, 2013; McDermott et al., 2013). Rova (2017) reported music and dance could cultivate a sense of social connections. Similarly, Mooren et al. (2017) found participants listening to catchy songs together helped to promote social connections. Hall et al. (2019) showed music influences motivation, self-image, and coping mechanisms related to problematic emotional states.

Music listening intervention delivered by MP3 players (e.g. iPods) and headphones can support a positive role for caregivers of people with dementia (Johnston et al., 2017). In a dementia unit, music listening effectively reduces a resident’s disruptive vocalization behaviors (Locke & Mudford, 2010). Music is personal. The literature has emphasized that music should be meaningful to the individual (McDermott et al., 2014). Mandzuk et al. (2018) provided personalized music in a hospital ward, the majority of older patients (90%) responded positively during their sessions with their personalized playlists. The staff in Locke & Mudford’s (2010) found music delivered by headphones was better than speakers because other residents were not forced to hear music they did not like. Hall et al. (2019) reported patients in mental health units could benefit from increasing positive engagement through music-based interventions to promote health and well-being. In more recent research on 17 older people with Alzheimer’s dementia, the Magnetic Resonance Imaging (MRI) scan showed widespread increases in functional connectivity in corticocortical and corticocerebellar networks following individualized favored music (King et al., 2019).

The study by Smeby (2016) demonstrated that music could help reduce stress, decrease antipsychotic use, soothe pain, and energize the body. A meta-analysis by Zhang et al. (2017) suggested that music helps treat responsive/aggressive behaviours among patients with dementia. Although research on music demonstrated effectiveness, there is a need for an investigation to understand how technology can be applied to enhance this non-pharmacological intervention in clinical settings, where care can be highly challenging. New technology, such as silent disco headphones, offers unique features that may support the needs of older patients with dementia.

## The Silent Disco Headphones

A new technology – the silent disco headphones (with a feature of noise cancellation) have been used in a large urban Canadian Hospital for more than 12 months since 2019. The wireless headphones have extended distance coverage for broadcasting music from an Ultra High Frequency (UHF)/Radio Frequency (RF) stereo transmitter. The external noise is silenced by sound wave cancellation. Rather than using a speaker system, music is delivered wirelessly by high-quality headphones. Through an AKG C3000 condenser microphone, a therapist may speak and sing to a group of patients with the headphones. Participants can customize their own audio experience by adjusting volume and switching between three channels of music (classical, jazz, and popular genre).

## Theoretical Grounding

This study is informed by the Technology Adoption Model (TAM), which has been widely used to explain how perceptions of a technology innovation affect technology’s eventual use (Davis, 1989). TAM explains users' motivation by three factors: perceived usefulness, perceived ease of use, and attitude toward use. The two concepts – perceived usefulness and ease of use – are viewed as having a considerable impact on the user's attitude. Taherdoost (2018) pointed out other external factors (such as the implementation process) should be considered in TAM as well. Caccia-Bava et al. (2006) argued that hospital culture and leadership’s capacity to innovate are key to technology adoption.

### The Research Team and Purpose

Our research team includes patient and family partners (JM, LP, LW), interdisciplinary clinicians (KD, MWH) and academic researchers (LH, HC). The hospital research institute funded the project to explore innovative ideas for patient care. LH is a nurse researcher at the hospital research institute and led the design of the study. LP and LW (family partner) performed data collection. LH led regular research meetings and facilitated team discussion in data analysis. The whole research team participated in the analysis. HC, a senior scientist, served as a mentor for the project. All members of the research team have a shared interest in the role of technology for supporting dementia care. The study's purpose was to examine the acceptability and feasibility of implementing the new technology of silent disco headphones in a geriatric hospital unit.

### Music Therapy and Music Listening

The music therapy group was implemented every Friday afternoon for one hour. Based on the guidelines for reporting music-based interventions by Robb et al. (2011), we provide details of the music group program in Table 1.

**Table 1.** Music Therapy Group Description.

A: Intervention rationale	The music group was expected to engage patients with both active and receptive music experiences. The primary goal was to help patients build resilience for health and wellness while stress, anxiety and emotional distress are common experiences during hospitalization.
B: Intervention content	The music therapist prepared a list of common songs (e.g., Somewhere over the rainbow) for the music group. Each patient took a turn to pick a song. The music therapist played guitar and sang the song to a microphone. Patients were invited to wear headphones to join the music group, sing along, and dance. Patients adjusted the volume of the music in their headphones according to their own preferences. The silent disco headphones helped the patients to focus on the music listening experience with the therapist while they are in the group with others.
C: Intervention delivery schedule	The music group sessions were one-hour long, every Friday afternoon
D: Interventionist	The accredited music therapist delivered all music group sessions.
E: Treatment	The music therapist applied a three-step procedure in all sessions. First, he began each session by greeting the patients. Second, he asked patients to take a turn to select a song. Patients were encouraged to take part in active activities such as singing and dancing. There were also passive activities, such as listening and watching others dancing in front of the group. Third, he invited patients to have conversations and reminisce between songs.
F: Setting	The music group was held in the common activity room on the unit. Patients could choose to wear the headphones to join the music group remotely in their own rooms or other unit areas.
G: Unit of delivery	Each music group program was delivered to 6–10 patients in a group

**Figure 1.**
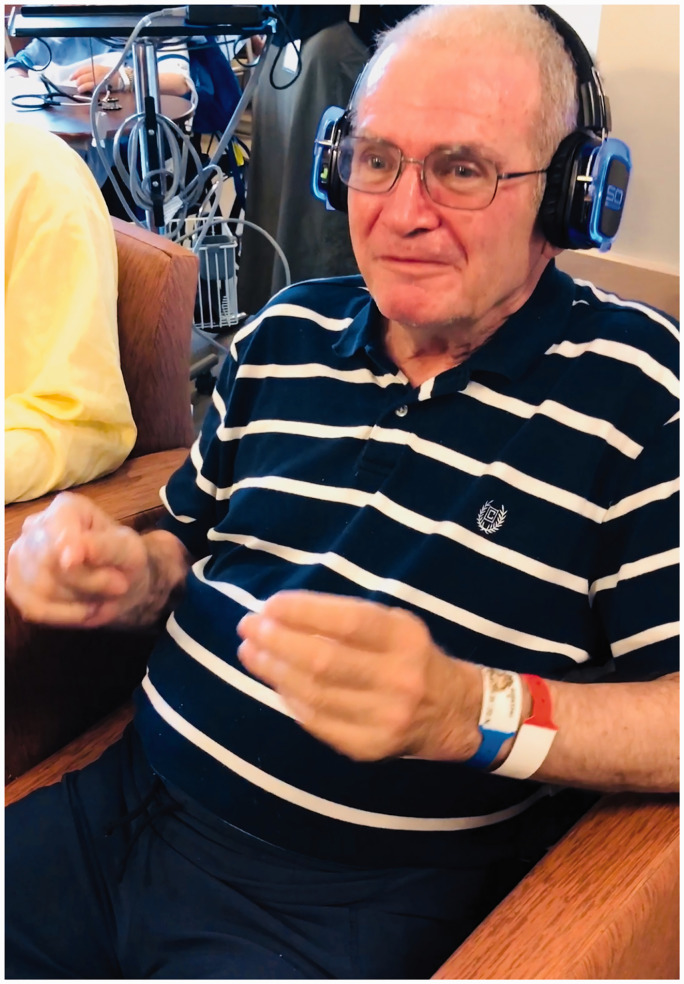
Ben Was Singing Rock Around the Clock With Finger-Snapping in a Music Group.

The accredited music therapist curated a pre-selected playlist of a variety of songs that older adults prefer. The playlist included common songs from the 1930s–1970s based on the music therapist's experience, who has provided older people music groups for 20 years. The music therapist conducted an assessment to find out the music preference of each patient. Patient music preferences were integrated along with the music therapist’s practice wisdom in selecting songs for the playlist. Patients also used the headphones individually to listen to music (outside music therapy) at different times, depending on their own needs. For example, staff have applied the headphones to provide positive distraction and help de-escalate behavioral symptoms. See Figure 1 for a patient participant using the headphones. Because silent disco headphones have never been studied in this population, we wanted to explore how the headphones can be used effectively with people with dementia in the clinical environment, what worked, what did not, why and under what circumstance.

## Methods

### Study Design

We employed a qualitative approach with video-ethnographic methods in data collection to explore the headphone intervention in a geriatric mental health unit. Qualitative research allows an in-depth and detailed investigation of how an intervention works or does not work in particular situations in a clinical setting and the complex interplay of factors that enable and hinder the intervention implementation (Thorne, 2015). Video ethnography (Neuwirth et al., 2012) enables capturing an accurate recording of interaction dynamics between people in the clinical context of occurrence. This naturalistic perspective allows for a realistic understanding of how interactions spontaneously emerge and evolve with the use of music by silent headphones in the hospital setting. Videos allow for scientific rigour by accurately recording the subtle, very complex proceedings in time and space for in-depth and repeated analysis (Knoblauch et al., 2015). Also, video data allow for review by researchers, staff, and patient and family partners in team analysis, which can help increase the scope of interpretation and compare perspectives to assist triangulation, which contributes to the rigour and accountability of the analysis and gaining a full picture of the studied phenomenon (Iedema et al., 2015).

### Research Questions

1. What are the patients’ experiences of using the silent disco headphone technology?
2. What are the staff’s perspectives on using the silent disco headphones? What enables and hinders them from using the headphones to deliver music in the hospital unit?

### Setting

The research was conducted at one older adult unit (19-bed) in a local hospital's mental health program, where the PI and the clinician co-authors work. The geriatric unit provides assessment and treatment related to neurocognitive disorders and mental health illnesses for older adults. The majority of the patients on the unit have a diagnosis of dementia and other co-morbidities. The length of stay varies from two weeks to six months or longer.

### Sample and Procedures for Recruitment

We used purposive sampling to recruit patient participants and convenient sampling to recruit staff participants across disciplines. Patients were recruited with assistance by nurses who knew the patients well in the unit. To maximize variations, we selected patients with different kinds of dementia, a wide range of ethnic backgrounds, males and females, etc. See Table 2 for the characteristics of patient participants. For staff recruitment, the unit educator sent an email to invite staff on the studied unit to participate. The email provided the research information and inclusion criteria. See Table 3 for the characteristics of staff participants.

**Table 2.** Descriptive Characteristics of Patient Participants (n = 10).

Patient characteristics	N (%)
Age (years)	
60–75	2 (20)
76–86	6 (60)
Older than 85	2 (20)
Gender	
Male	5 (50)
Female	5 (50)
Dementia	
Early stage	2 (20)
Middle stage	6 (60)
Late stage	2 (20)
Ethnicity	
Caucasian	7 (70)
South Asian	2 (20)
Black	1 (10)

**Table 3.** Descriptive Characteristics of Staff Participants (n = 10).

Staff characteristics	N (%)
Age (years)	
20–35	2 (20)
36–40	4 (40)
Older than 50	4 (40)
Gender	
Male	5 (50)
Female	5 (50)
Role	
Nurse	2 (20)
Care worker	3 (30)
Rehabilitation assistant	2 (20)
Occupational therapist	1 (10)
Recreation	1 (10)
Music therapist	1 (10)
Ethnicity	
Caucasian	4 (40)
South Asian	6 (60)

### Inclusion/Exclusion Criteria

Inclusion criteria for patient participants were: (a) inpatients staying in the studied unit, (b) able to speak English, (c) have a diagnosis of dementia or a mental health disorder, (d) have an interest in music, and (e) not in acute psychosis or suicidal. The inclusion criteria for staff participants were: (a) regular or casual nursing and allied health staff working on the studied unit, and (b) willing to help patients use the silent headphones for music.

### Data Collection

The combination of interviews and observation in ethnography enabled us to meaningfully look at the interaction between patients and the headphone technology in real-life clinical situations. Patient participants were observed and video-recorded 2–4 times on different times and days of the week, about 15–30 minutes each time. A researcher used a handheld camera to record patients’ responses with the use of headphones. Patient participants were interviewed right after the observations. We asked: what was your experience like about using the headphones for music? What did you like, and what did you not like? The interviews were conversational, lasting about 10–15 minutes. All interviews took place in the common activity area, where the music therapy program was provided. The interviews were video recorded and transcribed verbatim. Researchers wrote memos and field notes to document details about the staff and patient interactions, including non-verbal expressions, dance or bodily movement, and other emotional expressions. The field note also included researchers’ reflections in the field as part of the reflexive engagement with the field. Highlights of transcription, memos, field notes, and videos were brought to the research meeting to conduct team analysis with patient and family partners in the research team.

One focus group with ten staff participants was conducted in the nursing station of the unit. A patient partner (LW) facilitated the discussion, and the PI (LH) took notes. The discussion was audio-recorded and transcribed verbatim. Staff members were asked: What is your opinion and perspective of using silent disco headphones to support patients’ care? What enables and hinders you from using the silent disco headphones in the unit? What do you need to integrate music into routine patients’ care? The focus group lasted 40 minutes.

### Research Ethics

This study was approved by the university institutional review board and the hospital research institute. Informed written consent for participating in the study as well as for video recording was obtained from patient participants and their families. We treated consent as an ongoing process and carefully monitored verbal and non-verbal cues to the acceptability of the research activities (Black et al., 2010; Dewing, 2007). The patient consent form was initially obtained from each patient or his/her family. A family member signed the participant consent form when the patient participant was unable to read the research information and consent form. We obtained verbal assent before and during each interview and observation session and reminded patient participants about the purpose of the research and their right to withdraw at any time. We respected any behaviours indicating dissent. Participants were also provided with options of notetaking observations if they did not want to be video recorded. Also, we obtained an agreement with the family in the written consent for how the recordings would be performed, used and kept safe. Participants and families were invited to review video footage, and permission was sought for the specific use (e.g., for staff view in the local unit and presentation in academic conferences). Participants and families were offered options to use real names for acknowledgement of contributions or pseudonyms to protect anonymity. All staff participants provided written informed consent to enroll in the study. This article uses real names and pseudonyms as per agreement gained in the individual informed consent.

### Data Analysis

To examine the feasibility, we needed to understand how a new practice can be delivered in a real clinical setting to meet local needs. Visual data allowed us to see what was happening in the environment and how people responded to music in bodily ways. Patient interviews and staff focus group discussions were transcribed verbatim and analyzed using a descriptive interpretative analysis approach. Codes were applied to small data segments and grouped into categories and themes. Interpretive description is well suited to applied research that seeks practice applications through the lens of clinicians’ and patients’ experience (Thorne, 2016). Data collection and analysis occurred concurrently and in an iterative manner, so the early analysis informed the later data collection directions.

Clinicians who knew the patient participants provided additional contextual information to deepen the analysis. For example, one of the patient participants, Mr. Smith, could not stay seated in the music group; he needed to get up to walk. His wife visited him every afternoon and walked with him in the hallways. We then decided to give Mr. and Mrs. Smith headphones to join the group remotely while they walked. Our video data showed that Mr. Smith danced and showed a big smile in a later music group session. Staff in the focus group discussions provided more contextual information; they described and compared stories about when the headphones were used and when they were not used. The multiple perspectives helped generate a rich and detailed description of the patients’ responses to the headphones in various clinical situations.

The research team met weekly to discuss data collection, watch video data together and conduct team analysis. Research questions guided the development of our initial codes. We created a list of codes related to feasibility and acceptability to assign to the meaning units we identified in the transcripts. See Table 3 for the overview of the development of themes, example codes and quotes. Using NVivo 11, a software program for qualitative data analysis, we compared coded data across interviews, observation, and focus group data. Through a collaborative, participatory approach, we systematically categorized and compared data, ultimately clustered segments of data into empirically grounded themes that represented patients’ experiences and staff's perspectives of using the silent disco headphones in the clinical setting. In this way, evolving levels of thematic analysis were completed by moving between the whole and the specific (Thorne, 2016).

### Rigour

To ensure the rigour of this study, We have taken the following steps in this qualitative study according to the four principles in the Interpretive Description approach (Thorne, 2016):

- Epistemological Integrity: We acknowledge that our work relationship with the staff provided an advantage in recruiting and access to information (e.g., informal leaders among the staff, insider language and culture). Recognizing that greater familiarity can lead to researcher bias, reflexivity through team discussion regularly was practiced in weekly research meetings to protect epistemological integrity. Team reflection allowed us to discuss and compare our own assumptions and paid attention to how assumptions might influence our thinking and actions. Regular team reflexive meetings also help bring a shared awareness of the complexity of clinical situations and patient experiences (Hung et al., 2018).
- Representative Credibility: We used an inclusive approach to involve frontline clinicians and patient and family partners in the research team. The inclusion of staff, patient and family partners in the research team demonstrate both a recognition that knowledge exists beyond a single perspective or context and alignment with the research underpinning, which is to produce useful knowledge that informs clinical practice to improve patient care.
- Analytic Logic: To achieve credibility in analytic reasoning, our team analysis, including a diverse perspective from frontline clinicians, patient partner and family partners, made the process explicitly visible and transparent.
- Interpretive Authority: To enhance trustworthy illustrations, we provide a detailed description of the context, participants and methods of data generation.

## Results

Ten patients and ten staff members who participated in the study expressed their views about the feasibility and acceptability of using the silent disco headphones for music. See Tables 2 and 3 for patient and staff participants’ characteristics, respectively. The study's purpose was to examine the acceptability and feasibility of implementing the silent disco headphones in a geriatric hospital unit. Our analysis identified three themes that represented: (1) perceived usefulness, (2) ease of use, and (3) attitude to using technology. Table 4 provides an overview of the development of themes, codes and quotes.

**Table 4.** Overview of Themes, Codes, and Quotes.

Main themes	Code examples	Quote examples
Perceived usefulness	Patient experience – Feel good Patient experience – Joyful memory	Getting back into music just made me feel good. With these headphones, I can get into the mood right away. I love to sing. I love to dance. It was a joy. It brings back a memory: when I was a teenager, I used to go to the gay bar…
Perceived ease of use	Staff experience – It may seem more work Staff experience – Knowledge gap	The headphones can seem like more work. We do not have time for it. We are usually very busy. I don’t know how to get the music, what buttons to push to turn things on. Is it easy to make it work?
Attitude	Attitude – Acceptance Attitude change – See the patient in person-centred way	Every unit should have that. This is about supporting people to cope with stress and mental health It changed the way I see Alex [patient]. I was afraid of him because he seemed so aggressive. Now I know he has humor and a bit of fun too.

### Theme 1: Perceived Usefulness – “It Just Made Me Feel Good.”

This theme, perceived usefulness suggests that staff and patients finding the headphones useful. Many patient participants stated that staying at the hospital was a difficult experience, leading to emotional impact. Some patient participants felt sad and a sense of withdrawal, while others felt angry and frustrated. Many patient participants said they used to listen to music and played a musical instrument. Music was something that they enjoyed but did not always have access to when placed in the hospital setting. A patient participant, Merrilee, told us in the interview that the headphones were useful for her: “Getting back into music just made me feel good. With these headphones, I can get into the mood right away. I love to sing. I love to dance,” Our video data included moments of her singing along with other patients and dancing in front of groups. Her dance steps were impromptu, dynamic, creative, rhythmic, expressive and filled with fun.

The theme perceived usefulness also indicates the headphones were useful in terms of providing temporary pain relief. The music provided a positive diversion to distract the patient from her pain experience. The patient participant, Jane, got up to dance with the headphones on and smiled. During the study, Jane had a lot of pain, which limited her mobility and daily functions. The nursing staff told us, “she worries a lot about her health, illness symptoms, and pain.” Our observation and field notes found her spending a lot of time sitting alone. After a music therapy program, in the interview, Jane described, “these headphones are good, the sound is great. This is the second time I used the headphones, and I just feel good”. Then she went on to tell the researcher the kinds of music she likes and a list of artists, and memories of her past. The headphones were perceived by Jane as useful because they distracted her from pain. The music delivered by the headphones, as a mood enhancer, had taken Jane to places for positive and meaningful connections.

In the study, patients with early to later stages of dementia could participate in their ways. Participants adjusted the volume to fit their own needs by turning the volume knob on the right side of the headphones. Our video footage captured a few patient participants with a moderate stage of dementia used a finger to turn the volume knob to adjusted volume. For those with late-stage dementia, a staff helped them to set up a comfortable volume level. We saw the face of participants instantly light up as soon as they put on the headphones. For example, Ben, a patient, started to sing Rock Around the Clock with as much enthusiasm as Bill Haley despite the person sitting next to him was swearing. Ben just turned his volume up. His spirit brightened, and he had a joyful time. The music evoked not only emotions but also memories. When asked what it was like to use the headphones, Gary, a patient, responded:

It was wonderful. The best part was to be with you and others. We danced together to the music. It was a joy. It brings back a memory: when I was a teenager, I used to go to the gay bar and danced till two in the morning. It was so much fun.

### Theme 2: Perceived Ease of Use

In the beginning, some staff participants expressed concerns that helping the patients to set up the headphones could be an onerous task. They were clear that they would only implement the headphones if the headphones were easy to use. Jo, a care worker, commented: “The headphones can seem like more work. We do not have time for it. We are usually very busy.” After a pause, Jo went on to say, “but if we have quick access to the headphones [easy to grab from a convenient location], if it’s easy to connect to music, if it helps a patient to have a calm time, that could be less work, I think, for sure in the long run.”

Alice, the nurse leader, added, “It may seem like more work, but I see it saves time and makes our work easy. Some patients have a hearing impairment. This way, they can hear properly and then actually participate.” In our observation, we saw two patients with hearing impairment responded well to the therapist's verbal instruction through the headphones. One patient who could not hear normal conversation put the headphones over her ears and chirped, “wow, I can hear the music.” The benefit to people with hearing impairment is a potential benefit, which requires further investigation in future research. An occupational therapist remarked, “I see the patients were more focused on KD (Music Therapist) because they could hear him.”

In the focus group, a care worker raised concerns about their confidence in using headphone technology for music. Simon stated, “I don’t know how to get the music, what buttons to push to turn things on. Is it easy to make it work?” Another staff member, Mylra commented she found the headphones were easy to use and worked well for patients who were overstimulated in a noisy environment. A recreational staff Gail told a story: “the other day, Helen, a new patient, was agitated while she was pacing around the unit. Len, another patient, was listening to John Denver with a set of headphones. He invited Helen to dance to the song ‘Leaving on a Jet Plane’. It helped to settle Helen right away.” In our video data, the tension of Helen’s face disappeared. The effect of stress reduction and relaxation was evident as soon as Helen joined the dance. Another patient walked past them with loud swearing, but both Len and Helen did not notice. Staff participants commented that the bow and curtsey at the end of the dance indicated Len and Helen had an enjoyable experience. Maggie, a nurse, remarked, “See, the headphones can be used to de-escalate agitation and calm people down.” Positive stories were spread and influenced how other staff perceive the value of supporting patients to use the headphones in everyday care.

### Theme 3: Attitude

The comments we heard from staff across disciplines and nurse leaders showed acceptance in attitude. A care worker Genesis said, “They were more engaged in participating with singing and dancing. They looked so happy. I tried it myself. It takes away the external noise, and the sound is better – it gives you an immersive feeling. It blocks other unwanted noise like somebody might be yelling down the hall”. The music therapist explained, “yeah, it works because it doesn’t get lost in the acoustics of the room. But not everyone likes headphones. Some people can’t sit and stay with the music therapy program. It’s okay. They still can have the headphones on and wander through the unit. I think overall the music just sounds better and is more immersive”. Our field note showed a few family members and patients wore headphones and walked the hallways, joining the music program remotely in their own ways. After hearing the positive patient stories, a nurse leader Sheinoor commented: “I think every units should have the headphones. This is about supporting people to cope with stress and mental health. Some of our patients are very sound sensitive. The headphones allow people to listen to the music, blocking out the clinical world from overwhelming them. There is a lot of potential for benefits”.

Our observation suggested the headphones offer potential benefits for patients with various stages of dementia, including patients with later stages of dementia. The video data showed a few patients with severe dementia were able to interact meaningfully through embodied movement and non-verbal forms of communication. For example, a patient with late-stage Parkinson's dementia, Alex, who was considered as “resistive with care,” clapped his hands and hummed with the tune. The footage captured other patients’ subtle responses, such as tapping the foot, snapping fingers, and rocking the body side by side. Both leaders and staff were emotionally moved by the patients’ response when they watched the videos filmed in the unit. A care staff remarked, “It’s amazing to see those nonverbal expressions. It changed the way I see Alex [patient]. I was afraid of him because he seemed so aggressive. Now I know he has humor and a bit of fun too.’ Two family members asked the researchers to use the video of Alex to show everyone in the team the positive sides of Alex.

The recreation staff Gail also used the headphones to lead exercise groups, yoga and meditations. In an interview, Mathew (a patient) participated in a meditation program that offered a short mindfulness breathing exercise and nature music. Mathew said that the headphones offered him a respite and provided positive moments of joy. “It is an escape, a time away to rejuvenate,” The family partner LP said, “it does not have to be music; it can be white noise or a prayer.” A very experienced nurse, Ron agreed, “Yeah, it is useful when somebody gets up middle of the night or having trouble falling asleep. If you know music or a meditation message is what settles them, try it first before giving them medications.” Tara (a care worker) stressed it is important to know individual preference, “I think the headphones are cool. People may have different habits of using music. Just like Casey (a patient), he likes to lie in bed and put the headphones on, and just chill out.”

When a patient partner (LW) asked staff members about the barriers to the acceptance of the headphones, a few staff brought up issues of *not knowing how to connect*. Based on the staff focus group's feedback, a step-by-step tip sheet was posted to show how to connect the headphones with various playlists (e.g., classical, jazz, and popular) curated by the music therapist. The equipment was arranged in the central location (a common room outside the nursing station) so they were easy to grab. An iPad was mounted on the wall to make access to music more visible. *Leadership support* was identified as an essential enabling factor influencing the team’s attitude to adopting the new technology. Investment in materials and training are crucial for staff. For example, a nurse Ron, commented:

It is essential to recognize that this is something new, outside of our normal regular training. We are quite confident with medication management, but we were not educated about music headphones. It is nice that the leadership provided funding to buy the equipment. I would appreciate it if everyone gets support to learn how to use it.

## Discussion

This is the first study to explore the acceptability and feasibility of using the silent disco headphones technology to support older adults with dementia in a hospital setting. Our research generated a detailed and contextualized description of the patients’ responses to music delivered by the headphones in various clinical situations. Music memories helped patients to talk about their stories and engage in reminiscence activities. It was also evident that music delivered by the headphones offered respite or a positive distraction in stressful situations for some patients. The effects were immediate. It not only relaxed the patient but also stimulated the person in positive ways. Patient participants reported the music therapy program with the silent disco headphones brought positive benefits. Witnessing the positive effects on patients influenced the staff’s perception of how music could be used in the clinical setting to support patients’ well-being. Our findings are consistent with current literature regarding using music to help people with dementia and mental health disorders (Aalbers et al., 2017; Carr et al., 2013). Our study contributes to the literature by adding new knowledge about applying the silent disco headphone technology to deliver music and other potential programs.

Our study indicates promising effects of silent disco headphone technology for the unique needs of patients with dementia. The personalized control of volume and noise cancellation are helpful to filtering out unwanted environmental noise. The headphone connection with the wireless UHF/RF technology made it easier for the patients to focus and hear the therapist/staff. In the interviews, participants demonstrated that they enjoyed expressing their memories and emotions in the music group. For patients, music is a powerful and meaningful way of self-expression. People with dementia and mental health conditions often face problems of social stigma and social exclusion. Opportunity for social engagement and participation is vital for affirming a sense of self and community belonging. Due to disabilities and functional declines associated with dementia, older adults may internalize the stigmatizing views and withdraw from social activities. Dementia itself has already put people living with the disease at high risk of social isolation and loneliness. Although the infection prevention procedures during the COVID-19 pandemic are necessary, they have harmful effects and exacerbated social isolation and loneliness. The silent disco headphones can provide safe and remote access to music therapy group, which can be a helpful distraction from negative thoughts, anxiety, and loneliness. Music is more than a tool to manage agitation and behavioural symptoms. As suggested by Kontos (2019), music activities can enrich people's lives with dementia and promote social inclusion. Social inclusion is an experience and a feeling of belonging, which helps to reduce social isolation and loneliness. In this study, a sense of shared enjoyment can be noticed in music group. As Kontos (2019) described, when people enjoy an activity together with a song, there is a sense of self and inclusion, which can be vitally important to people with dementia.

For the lessons learned in this study, staff have identified knowledge gap as a barrier. Providing time for training support is important to overcome barriers to the knowledge gap. Leadership support has been identified as an enabling factor for the implementation of the headphones. The nursing leader and music therapist's active involvement helped the team understand the how and why of using the headphone to improve patient care. The patient stories and positive narratives told by local leaders encouraged many staff members in the team to try the new practice. As Caccia-Bava et al. (2006) state, current literature neglected local leaders' vital role in technology adoption. Future research should pay more attention to leadership support.

The growing evidence demonstrates that practice development requires a supportive environment for team learning and leadership support (Dewar & MacBride, 2017; Hung et al., 2017). Yet, inadequate evidence is available to inform how such a positive learning culture can be created to support technology adoption in hospital units. Moving forward, more attention should be paid to target practice development at the local clinical level (Chadder, 2019; Manley et al., 2011). In our study, the patient stories demonstrated in the videos were vital to motivate staff’s acceptance of technology adoption. This shows that demonstrating the valuable benefits of the new technology could alter the attitude of the staff.

Finally, this study highlights the salience of involving stakeholders in practice development. The narratives of patient participants and frontline staff supported the adoption of the new technology in local practice. Future research should involve patient partners and frontline staff in research to understand better what facilitates technology adoption and spreading innovation.

## Strengths and Limitations

One of the crucial strengths of this study is the meaningful involvement of patient and family partners in research. Our patient and family partners were involved early at the beginning of the study and throughout the research. They took on an active role in data collection, such as filming videos and facilitating staff focus groups. The regular weekly meetings kept all team members actively connected for research activities and team reflection. Together, we provided a rich interpretation of patients’ responses and staff perspectives in employing this novel practice. This study is limited to exploring patients' spontaneous responses as we did not study the long-term effects.

### Implications for Practice

This research extends on previous evidence highlighting the salience of involving users (hospital leadership, frontline staff, patients and families) in the planning and implementing of new practice in formal care settings (Hung, 2017). Project leads should include stakeholders early and in each stage of the development, from design, planning, implementation to evaluation. For adoption success, it is vital to understand the local culture, what people value, competing priorities, barriers, and what possible strategies would work to meet local needs. Knowing how frontline staff acquire, develop, and practice new technology skills is critical to gain acceptability. Successful integration of technology in practice requires perceived usefulness, perceived ease of use and acceptance in the attitude. This study suggests staff are willing, with support and resources, to adopt the silence disco headphone in practice. Our findings support the use of silence disco headphones as a promising means of improving patients' well-being. Leadership support (e.g., adapting the environment to make the equipment easy to access, providing resources and staff training) can enable staff to overcome challenges to adoption. As we are adapting to the new infection prevention procedures due to the COVID-19 pandemic, the silent disco headphones can provide safe and remote access to music therapy group in care settings. Future research should further investigate the effectiveness of remote music group in patient outcomes such as reducing loneliness.

## Conclusions

Our findings demonstrated that the silent disco headphone technology is feasible and acceptable to be implemented in the geriatric hospital unit. Patient participants reported that the use of silent disco headphones brought valuable benefits. The positive patients’ responses to the headphones influenced the staff’s view of how music could be used in the clinical setting to support patients’ well-being. The findings can be used to inform future research and practice development in clinical settings.
